# Immunologic Responses to *Vibrio cholerae* in Patients Co-Infected with Intestinal Parasites in Bangladesh

**DOI:** 10.1371/journal.pntd.0000403

**Published:** 2009-03-31

**Authors:** Jason B. Harris, Michael J. Podolsky, Taufiqur R. Bhuiyan, Fahima Chowdhury, Ashraful I. Khan, Regina C. LaRocque, Tanya Logvinenko, Jennifer Kendall, Abu S. G. Faruque, Cathryn R. Nagler, Edward T. Ryan, Firdausi Qadri, Stephen B. Calderwood

**Affiliations:** 1 Division of Infectious Diseases, Massachusetts General Hospital, Boston, Massachusetts, United States of America; 2 Department of Pediatrics, Harvard Medical School, Boston, Massachusetts, United States of America; 3 International Centre for Diarrhoeal Disease Research, Dhaka, Bangladesh; 4 Department of Medicine, Harvard Medical School, Boston, Massachusetts, United States of America; 5 Biostatistics Research Center, ICRHPS, Tufts Medical Center, Boston, Massachusetts, United States of America; 6 Department of Immunology and Infectious Diseases, Harvard School of Public Health, Boston, Massachusetts, United States of America; 7 Department of Microbiology and Molecular Genetics, Harvard Medical School, Boston, Massachusetts, United States of America; Uniformed Services University, United States of America

## Abstract

**Background:**

Infection with intestinal helminths is common and may contribute to the decreased efficacy of *Vibrio cholerae* vaccines in endemic compared to non-endemic areas. However, the immunomodulatory effects of concomitant intestinal parasitic infection in cholera patients have not been systematically evaluated.

**Methods:**

We evaluated *V. cholerae*-specific immune responses in a cohort of patients with severe cholera. 361 patients completed 21 days of observation and 53 (15%) had evidence of a concomitant intestinal parasitic infection based on direct microscopy. Although there were no significant differences in the vibriocidal or lipopolysaccharide (LPS)-specific immune responses to *V. cholerae*, helminth-infected cholera patients had decreased fecal and serum IgA immune responses to the B subunit of cholera toxin (CTB) as well as a more modest decrease in serum IgG response to CTB. These findings remained significant for all classes of helminth infection and when controlling for potential confounding variables such as age and nutritional status. Although we hypothesized the differential effect on CTB and LPS immune responses was due to T-cell-dependent immunomodulatory effects of helminth infection, we did not find additional evidence to support a classic Th1 or Th2 polarization of the immune response to *V. cholerae* infection related to parasite infection.

**Conclusions/Significance:**

The finding that helminth infection has a profound association with the mucosal humoral immune response to *V. cholerae* has implications for the development of protective immunity in cholera-endemic areas and provides an additional basis for deworming programs in cholera-endemic areas. Additional studies, including further characterization of the role of T cells in the immune response to human *V. cholerae* infection and the development of an animal model of co-infection, may provide additional insight into the mechanisms underlying these findings.

## Author Summary

*Vibrio cholerae* causes cholera, a severe diarrhea that may lead to fatal dehydration if not treated. Cholera occurs mostly in impoverished areas where there is poor sanitation and intestinal parasites are also common. However, little is known about the relationship between intestinal parasites and cholera. To learn about how parasites affect the immune response to *Vibrio cholerae*, this article describes 361 patients with cholera, including 53 who had intestinal parasitic infection. We found that cholera patients with parasitic worms had decreased antibody response to cholera toxin. The decrease was greatest in IgA antibodies, which are secreted in the intestine. However, patients with worm infection did not have a difference in their immune response to lipopolysaccharide, a sugar-based molecule that is important for immunity. These different effects on the immune response to cholera toxin and lipopolysaccharide could be explained by the effect of parasitic infection on CD4+ T cells, a type of cell that influences the development of the antibody response to proteins such as cholera toxin but may not always influence the response to sugar-based molecules. The finding that worm infection is associated with decreased immune responses to cholera provides an additional reason for deworming in cholera-endemic areas.

## Introduction

*V. cholerae* is a major cause of diarrhea globally and is estimated to cause five million cases of cholera annually, resulting in more than 100,000 deaths [1]. The vast majority of cases occur in developing countries. Cholera is endemic in Bangladesh, with an approximate incidence of 200 cases/100,000 individuals per year, where the majority of fatal cases occur in young children [2],[3]. Intestinal parasitic infections are also common among children in developing countries, and in rural Bangladesh, it is estimated that 80% of children are infected with the intestinal helminth *Ascaris lumbricoides* [4].

Despite the demographic overlap, little has been done to evaluate the epidemiologic, clinical and immunologic aspects of co-infection with *V. cholerae* and intestinal parasites. Hospital-based surveillance in Kolkata, India demonstrated that among children ages 2 to 10 presenting with acute diarrheal illness with *V. cholerae* infection, 30% had evidence of intestinal parasitic infection on direct stool examination, although the distribution of specific parasites was not reported [5]. A 30% prevalence of concomitant parasitic infection was also reported in *V. cholerae* infected patients in Kathmandu [6]. Whether intestinal parasitic co-infection modifies the clinical manifestations of *V. cholerae* infection in human is unknown; mice co-infected with the intestinal stage of *Trichinella spiralis* have a markedly reduced capacity to absorb fluid secreted in response to cholera toxin [7].

Co-infection with intestinal parasites may affect the immune responses to *V. cholerae* infection. In general, symptomatic infection with *V. cholerae* induces long-lasting protective immunity and the majority of patients with cholera develop robust humoral and mucosal immune responses. The best studied of the antibacterial immune responses to *V. cholerae* is the serum vibriocidal antibody, which is a complement-dependent bactericidal antibody directed primarily against *V. cholerae* LPS [8]. In Bangladesh, vibriocidal antibodies increase with age and are associated with protection from infection with *V. cholerae* [9]–[11]. Antitoxin immune responses are primarily directed to the B subunit of cholera toxin (CTB) [12], and levels of CTB-specific IgA antibodies are associated with protection from cholera independently of the vibriocidal antibody titer [13].

Although the effects of concomitant parasitic infection on the immune response to natural cholera have not been systematically evaluated, it is hypothesized that intestinal helminth infection may contribute to the decreased efficacy of live-attenuated *V. cholerae* vaccines in endemic compared to non-endemic areas. The live-attenuated *V. cholerae* vaccine strain, CVD103-HgR, was created by deleting the majority of the gene encoding the cholera toxin A subunit (CTA) [14]. North American and European adult volunteers ingesting one dose of the vaccine showed vibriocidal seroconversion in >90% of recipients, but only 16% of children from an endemic area of Indonesia demonstrated seroconversion [15]. CVD103-HgR showed 80% protective efficacy against diarrheal disease when U.S. volunteers were challenged with El Tor *V. cholerae* O1 [16]. However, in a large, randomized, placebo-controlled, double-blinded field trial in a cholera-endemic area of Indonesia, CVD103-HgR had a protective efficacy of only 14% [15]. To address the question of whether concomitant parasitic infection might explain this discordance, Cooper et. al. randomized 233 Ecuadorian children with *A. lumbricoides* infection to receive albendazole or placebo followed by CVD 103-HgR. Among those who completed the study, there was a trend towards higher vibriocidal seroconversion in albendazole recipients (30% vs 16%, P = 0.06) [17]. In a subset of individuals from this study, those treated with albendazole had an increased IL-2 response to stimulation of peripheral blood mononuclear cells by the B subunit of cholera toxin (CTB), suggesting an improved Th1-type response in children cleared of helminth infection prior to vaccination [18].

Although these data demonstrate that concomitant parasitic infection dampens the immune response to CVD103-HgR, it remains unclear whether helminth infection also affects the protective immune responses following cholera or other cholera vaccines. To better understand how preexisting infection with intestinal parasites affects the response to cholera, we evaluated the results of a prospective, observational study of immunologic responses to *V. cholerae* in patients with acute severe dehydrating diarrhea.

## Methods

### Study design and subject enrollment

The hospital of the International Centre for Diarrhoeal Disease Research, Bangladesh (ICDDR,B) provides care for more than 100,000 patients annually, including over 20,000 cholera patients, the majority of whom are residents of Dhaka city. Cases presenting to the hospital with severe acute watery diarrhea were eligible for inclusion in this study if their stool cultures were subsequently positive for *V. cholerae*, if they were older than 6 months, and if they were without significant co-morbid conditions. All patients had stool and blood specimens collected on the day after presentation (termed study day 2), to allow for clinical stabilization and confirmation of a positive stool culture result. Follow-up blood samples for immunologic analysis were obtained again on study days 7 and 21. Informed written consent for participation in this research was obtained from participants or their guardians. The human experimentation guidelines of the U.S. Department of Health and Human Services were followed in the conduct of this research, which was approved by the Institutional Review Board of the Massachusetts General Hospital and the Research and Ethical Review Committees of the ICDDR,B.

### Microbiologic studies

All cases of cholera were confirmed by culturing stool for *V. cholerae* on taurocholate-tellurite-gelatin agar (TTGA). After overnight incubation of plates, serological confirmation of suspected *V. cholerae* colonies was carried out by slide agglutination [19],[20]. In patients with confirmed cholera, stools were examined for intestinal parasites using direct microscopy. Two slides were prepared for each cholera patient. For rice-water stools, a drop of stool was placed directly under a cover slip, and for more solid stools, a thin preparation was prepared using approximately one to two grams of stool resuspended in normal saline. A second slide was prepared using 1% Lugol's iodine. Slides were prepared using a 22 mm cover glass, and the entire film was examined for parasites using light microscopy.

### Measurement of vibriocidal and cholera specific antibody responses in serum and fecal extracts

Vibriocidal antibody assays were performed with methodology previously described, using guinea pig complement and the homologous serotype of *V. cholerae* O1 or O139 as the target organism [21]. Serum and fecal antibodies specific to CTB, and LPS were measured by kinetic ELISAs using methods described previously [22]. 96-well microtiter plates were coated with either purified LPS (250 ng/well), or sequentially with GM1 ganglioside (100 ng/well) followed by recombinant CTB (50 ng/well) (gifts of A.M. Svennerholm). One-hundred fold dilutions of serum samples were used to measure total IgG and IgA to CTB and LPS, while forty-fold diluted serum samples were used for CTB specific IgG subclass antibody detection and eight-fold dilutions for IgE.

For measuring total IgG and IgA responses, horseradish peroxidase-conjugated secondary antibodies were applied at a 1∶1000 dilution (Jackson Laboratories, Bar Harbor, Maine). Biotinylated mouse monoclonal anti-human subclass-specific IgG1 (clone 8c/6-39), IgG2 (clone HP6014), IgG3 (clone HP-6050), and IgG4 (HP-6025) conjugates (Sigma) and IgE (clone G7-26) conjugate were added at a 1∶2000 dilution in PBS-Tween and incubated for 1 hour at 37°C. A 1∶4000 dilution of streptavidin-conjugated horseradish peroxidase (Thermo-Scientific) was used as the detection enzyme for biotinylated monoclonal antibodies. After incubation and washing, the plates were developed using 0.1% ortho-phenylene diamine (Sigma, St. Louis, Missouri) in 0.1 M sodium citrate buffer with 0.1% hydrogen peroxide, and optical densities (OD) were read kinetically at 450 nm for 5 minutes at 19-s intervals. Antigen specific-IgA levels in fecal extracts were expressed as a fraction of total IgA in fecal extracts, which was determined by ELISA using an IgA standard (1 mg/ml) derived from human colostrum, as described previously [22]. ELISA results were normalized using a pooled convalescent standard prepared from patients recently recovered from cholera.

### Statistical analysis

Analyses were performed using Stata version 9.0 (Stata Corporation, Inc., College Station, Texas), and R version 2.7.0 (http://cran.r-project.org/). Demographic, clinical and immunologic characteristics of the parasite-infected cases were compared with characteristics of the cases without evidence of parasitic infection using the Students' t-test or Chi^2^ statistic for binary characteristics. Multivariate analysis of baseline characteristics, the convalescent vibriocidal responses, and the CTB-specific antibody responses of cases was performed using multiple linear regression with all possible interaction terms for each variable, and the criteria for inclusion/exclusion of variables and interaction terms in the final model based on goodness of fit as determined by a decrease in Akaike information criterion. All reported p values are two-tailed.

## Results

### Characteristics of the cohort

400 patients with acute watery diarrhea due to *V. cholerae* O1 and O139 were enrolled between January 2001 and June 2006. Of the 400 patients enrolled, 361 completed (90%) at least 21 days of follow-up. Of the 400 enrolled patients, 58 (15%) had a concomitant parasitic infection detected by stool microscopy. Of 361 patients who completed immunologic follow-up, 53 (15%) of these patients had a concomitant parasitic infection. Of the 39 patients that were lost to follow up, 5 (15%) also had a concomitant parasitic infection; thus there did not appear to be a difference in attrition between parasite infected and non-infected cholera patients in this study. The type of parasitic infection in the 361 patients who completed follow-up is shown in Table 1. Helminth infection was observed in 41 (77%) of the 53 parasite-infected cholera cases, with *A. lumbricoides* being the most common parasitic infection detected in the cohort.

**Table 1:** Distribution of parasitic infections in a cohort of cholera patients in Dhaka, Bangladesh.

Category	Number	Percent of Cohort
All patients	361	-
Non-infected	308	85%
Parasite infected	53	15%
Helminths		
Any helminth	41	11%
Ascaris	35	10%
Hookworm	7	2%
Trichuris	8	2%
Protozoa		
Any protozoa	12	3%
Entamoeba	4	1%
Giardia	10	3%
Cryptosporidium	1	<1%
Multiple parasites	11	3%

Additional demographic and clinical data on the cholera patients are presented in Table 2. The vast majority of patients in this study had severe disease. Cholera patients presented to the clinical center an average of 17 hours after the onset of diarrhea, and over 90% had severe dehydration on presentation. There was no difference in severity or duration of illness in parasite-infected verses non-infected individuals. Similarly, there was no difference in the distribution of blood group phenotype, gender, or infecting serogroup of *V. cholerae* (O1 or O139) in the parasite infected versus non-infected patients.

**Table 2:** Characteristics of parasite-infected and non-infected cholera patients*.

	Entire Cohort	Parasite- Infected	No Parasite	P Value	Helminth Infected	No Helminth	P Value	Protozoa Infected	No Protozoa	P Value
	N = 361	N = 53	N = 308		N = 41	N = 320		N = 12	N = 349	
**Demographic Data**										
Mean Age	23 (±15)	18 (±11)	24 (±15)	0.009	18 (±12)	23 (±15)	0.02	18 (±8)	23 (±15)	0.28
Female	196 (54%)	34 (64%)	162 (53%)	0.12	27 (66%)	170 (53%)	0.12	7 (58%)	190 (54%)	0.78
**Clinical Data**										
Duration of Illness	17 (±14)	18 (±12)	17 (±14)	0.63	18 (±10)	17 (±14)	0.97	22 (±12)	17 (±14)	0.22
Severe dehydration	326 (91%)	48 (91%)	278 (91%)	1.0	36 (88%)	291 (91%)	0.52	12 (100%)	315 (90%)	0.25
Height-for-Age (Z)**	−1.97 (±1.13)	−2.00 (±1.12)	−1.97 (±1.14)	0.77	2.06 (±1.13)	1.96 (±1.13)	0.70	1.90 (±1.17)	1.98 (±1.13)	0.89
**Microbiologic**										
*V. cholerae* O139	57 (16%)	8 (15%)	49 (16%)	0.88	6 (15%)	51 (16%)	0.83	2 (17%)	55 (16%)	0.93
**Blood Group**										
O	147 (40%)	22 (41%)	124 (40%)	0.88	16 (39%)	130 (41%)	0.83	6 (50%)	140 (40%)	0.50
A	86 (24%)	11 (21%)	75 (24%)	0.56	10 (24%)	76 (24%)	0.94	1 (8%)	85 (24%)	0.20
B	103 (29%)	14 (26%)	89 (29%)	0.70	10 (24%)	93 (30%)	0.52	4 (33%)	99 (28%)	0.71
AB	25 (7%)	6 (11%)	19 (6%)	0.18	5 (12%)	20 (6%)	0.16	1 (8%)	24 (7%)	0.85

*: Mean values are reported with ±SD, and numbers of patients are reported with proportion of patients in each category, corresponding to the denominator (N) indicated in the column heading.

**: Height-for-Age (Z) reported for children and adolescents ≤18 years.

The average age of the cholera patients enrolled in this study was 23 years. There was a significant difference in the mean age of parasite-infected (18 years) versus non-infected patients (24 years). An equivalent age difference was seen in the helminth- and protozoa-infected patients, but this difference only reached statistical significance for the larger helminth infected group. We anticipated that parasite-infected children might have more stunted linear growth, but no appreciable difference in height-for-age was observed between the two groups.

### *V. cholerae*-specific immunologic responses in parasite infected versus non-infected cholera patients

The acute and convalescent antibody responses to *V. cholerae* antigens of parasite-infected and non-infected patients are presented in Table 3. No significant differences were observed in the vibriocidal antibody response or in the LPS-specific antibody responses to severe *V. cholerae* infection. Only one (1.8%) parasite infected patient had less than a four-fold increase in their vibriocidal antibody, while four (1.3%) non-parasite infected patients had less than a four-fold increase in their vibriocidal antibody (P = 0.73).

**Table 3:** Acute and convalescent *V. cholerae*-specific immune responses of parasite infected and non-infected cholera patients.

Immunologic Variable	Day	Entire Cohort	Parasite Infected	No Parasite	P Value	Helminth Infected	No Helminth	P Value	Protozoa Infected	No Protozoa	P Value
		N = 361	N = 53	N = 308		N = 41	N = 320		N = 12	N = 349	
**Serum Response** *											
Vibriocidal response	2	5.8 (±2.5)	6.2 (±2.5)	5.8 (±2.5)	0.27	6.2 (±2.3)	5.8 (±2.6)	0.38	6.3 (±3.3)	5.8 (±2.5)	0.51
	21	11.2 (±1.9)	11.1 (±2.5)	11.2 (±1.8)	0.84	11.2 (±1.9)	11.2 (±1.9)	0.81	10.7 (±4.2)	11.2 (±1.8)	0.42
CTB IgA	2	4.3 (±1.2)	4.0 (±1.4)	4.4 (±1.2)	0.06	4.1 (±1.2)	4.3 (±1.2)	0.17	3.9 (±1.8)	4.3 (±1.2)	0.23
	21	6.2 (±1.2)	5.6 (±1.4)	6.3 (±1.1)	<0.0001	5.5 (±1.4)	6.3 (±1.2)	<0.0001	6.0 (±1.4)	6.2 (±1.1)	0.29
CTB IgG	2	6.6 (±1.0)	6.6 (±0.8)	6.6 (±1.0)	0.90	6.6 (±0.8)	6.6 (±1.0)	0.87	6.6 (±0.9)	6.5 (±1.0)	0.58
	21	8.2 (±0.8)	7.9 (±0.8)	8.2 (±0.8)	0.01	7.9 (±0.8)	8.2 (±0.7)	0.01	8.2 (±0.5)	8.2 (±0.8)	0.90
LPS IgA	2	4.1 (±1.3)	4.2 (±1.3)	4.1 (±1.3)	0.70	4.3 (±1.2)	4.1 (±1.3)	0.40	3.8 (±1.7)	4.1 (±1.3)	0.47
	21	6.0 (±1.7)	5.9 (±1.6)	6.0 (±1.7)	0.59	5.9 (±1.6)	6.0 (±1.7)	0.79	5.7 (±1.7)	6.0 (±1.7)	0.55
LPS IgG	2	6.2 (±0.9)	6.1 (±0.9)	6.2 (±0.9)	0.36	6.1 (±0.9)	6.2 (±0.9)	0.49	6.1 (±1.1)	6.2 (±0.9)	0.54
	21	7.6 (±0.9)	7.5 (±0.9)	7.6 (±0.9)	0.22	7.5 (±0.9)	7.6 (±0.9)	0.44	7.3 (±1.0)	7.6 (±0.9)	0.29
**Fecal IgA Response** **											
Fecal CTB IgA	2	3.7 (±1.4)	2.8 (±1.6)	3.9 (±1.3)	0.0004	2.6 (±1.5)	3.8 (±1.3)	0.0004	3.3 (±1.7)	3.7 (±1.4)	0.41
	21	4.1 (±1.9)	4.3 (±1.9)	4.1 (±1.9)	0.50	4.4 (±1.9)	4.1 (±1.9)	0.45	4.1 (±2.0)	4.1 (±1.9)	0.96
Fecal LPS IgA	2	1.4 (±1.4)	1.3 (±1.6)	1.4 (±1.4)	0.80	1.4 (±1.2)	1.4 (±1.2)	0.40	2.2 (±2.1)	1.3 (±1.3)	0.07
	21	2.1 (±1.8)	2.2 (±1.8)	2.1 (±1.8)	0.73	2.5 (±1.9)	2.1 (±1.7)	0.36	1.6 (±1.3)	2.1 (±1.8)	0.44

*: Serum vibriocidal titers are expressed as log2 inverse antibody titers. CTB and LPS antibody levels are expressed as log2 normalized ELISA units.

**: Fecal IgA antibody levels are expressed as log2 normalized ELISA units, adjusted for total fecal IgA.

Parasite-infected individuals had several significant differences in their immune responses to the CTB antigen (Table 3). These included: 1. a lower level of fecal CTB-specific IgA antibodies on the day after presentation (a difference of 1.1 log_2_, P = 0.0004). 2. a lower convalescent serum IgA response to CTB (a difference of 0.8 log_2_, P<0.0001), and 3. a modestly lower convalescent serum IgG response to CTB (a difference of 0.3 log_2_, P = 0.03).

To evaluate whether these differences in the immune responses to CTB were unique to helminth or protozoal infection, we performed a sub-analysis, as shown in Table 3. Restricting the analysis to helminth-infected versus non-infected patients revealed a larger and more statistically robust difference for all three significant findings, while there was no discernable difference in CTB responses in protozoa-infected versus non-infected patients.

**Figure 1:**
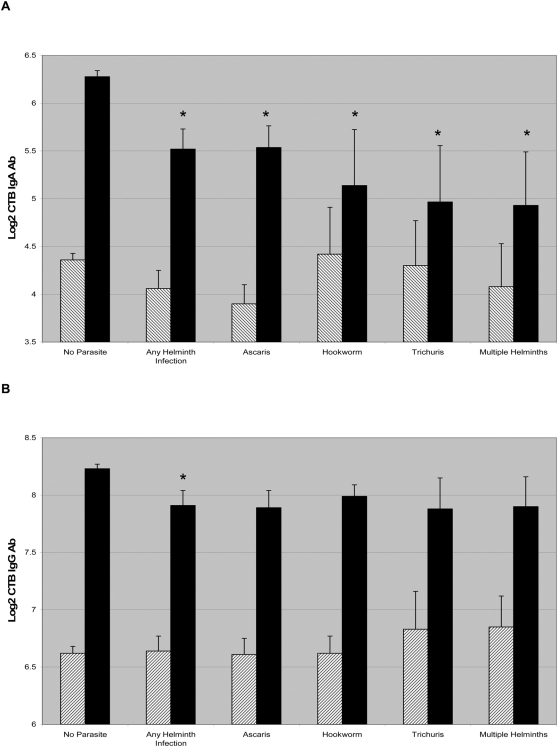
CTB specific IgA and IgG responses are shown in cholera patients across classes of helminth infection (A and B, respectively). The hatched bars indicate the mean log2 transformed CTB titer on day 2, and the dark bars indicate the titer on day 21 and error bars indicate the standard error of the mean. An asterisk denotes a statistically significant difference between non-infected and infected individuals. All classes of helminth infected patients had significantly lower CTB IgA immune responses on day 21 compared to non-helminth infected controls.

Because the immunologic differences in responses to *V. cholerae* were restricted to helminth infection, we compared acute and convalescent CTB antibody titers in patients with different helminth infections, as shown in Figure 1. Individuals infected with all classes of intestinal helminths had significantly decreased CTB-specific IgA responses, with patients infected with multiple helminths having the most impaired CTB-specific IgA response. Again, we observed a modest but similar decrease in the CTB-specific IgG response across classes of geohelminths, although this finding only reached statistical significance for the aggregate of all helminth-infected patients.

### Multivariate analysis of selected immune responses in cholera patients

The vibriocidal antibody and the serum CTB-specific IgA antibody are the only known independent immunologic predictors of protection against *V. cholerae* O1 infection, and are thus important markers of protective immunity to cholera [13]. To evaluate whether the associations between helminth infection and immune responses to *V. cholerae* might be due to confounding, we conducted a multivariate regression analysis of these immune responses including age, gender, and blood group for the *V. cholerae* O1 serogroup as shown in Table 4.

**Table 4:** Multivariate model of the association of helminth infection with convalescent immune responses in cholera patients.

Factors associated with convalescent vibriocidal antibody titer.*	Regression Coefficient	Standard Error	P
(Intercept)	12.02	0.229	<0.0001
Helminth Infection	−0.208	0.308	0.50
Age (years)	−0.017	0.007	0.02
Male gender	−0.549	0.199	0.01
Blood group O	0.407	0.199	0.04

Factors associated with convalescent CTB IgA antibody titer.**	Regression Coefficient	Standard Error	P
(Intercept)	6.46	0.156	<0.0001
Helminth Infection	−0.856	0.207	<0.0001
Blood group O	−0.168	0.238	0.48
Age (years)	−0.016	0.006	0.01
Blood group O: Age (years)	0.020	0.009	0.03

*: After selection all interaction terms were excluded from the model.

**: After selection gender and all interaction terms other than the interaction between blood group O and age were excluded from the model.

Multivariate analysis demonstrated that the relationship between helminth infection and the CTB-specific IgA response was highly statistically significant and independent of the other variables included in the model (regression coefficient −0.86, P = 4.8*10^−5^). In addition, to examine whether nutritional factors played a role in mediating the relationship between helminth infection and the immune responses, separate sub-analyses were performed on children less than 18 years old (for whom height-for-age data (N = 123) were available) and on patients in whom serum retinol data (N = 128) were available. Even in these sub-analyses, helminth infection remained a significant predictor of the convalescent IgA response to CTB (regression coefficient −0.81, P = 0.008, and regression coefficient −0.93, P = 0.004 respectively), demonstrating that the association between helminth infection and the convalescent CTB response was independent of possible interactions with nutritional status. However, no significant relationship between helminth infection and vibriocidal antibody levels (P = 0.500) was found.

### Immunoglobulin subclasses and cytokine responses in parasite-infected versus non-infected patients

Although CTB-specific IgG responses were significantly decreased in helminth infected individuals, the magnitude of this difference was less than that observed for CTB-specific IgA. To evaluate whether the association of helminth infection with the CTB immune response was specific to certain IgG subclasses, and to assess whether this association had a Th1 or Th2 polarization, we evaluated the IgG subclass responses of 34 helminth-infected cases and non-infected controls matched by age (within 2 years), gender, and infecting serogroup. These results are presented in Table 5. Compared to the controls, the helminth-infected patients had lower CTB-specific IgG responses of all subclasses, although this only reached statistical significance for IgG1 (a difference of 0.8 log_2_, P = 0.03). Among 12 matched pairs with sufficient sample to measure CTB-specific IgE specific responses, no difference was observed between cases and controls.

**Table 5:** CTB-specific immunoglobulin subclass responses in helminth-infected and matched non-infected cholera patients.

Immunoglobulin	Day	Cases (N = 34)	Controls (N = 34)	P
Total IgG	2	6.6 (±0.8)	6.8 (±1.0)	0.34
	21	7.9 (±0.8)	8.4 (±0.8)	0.03
IgG1	2	3.9 (±1.1)	4.1 (±0.9)	0.30
	21	7.0 (±1.5)	7.8 (±1.8)	0.03
IgG2	2	2.9 (±1.2)	2.9 (±1.6)	0.82
	21	6.4 (±1.5)	6.7 (±1.4)	0.36
IgG3	2	5.6 (±1.2)	5.1 (±1.2)	0.09
	21	7.9 (±1.5)	8.2 (±1.3)	0.36
IgG4	2	4.6 (±1.5)	4.5 (±1.5)	0.57
	21	7.2 (±1.8)	7.5 (±2.0)	0.33
Total IgE*	2	2.7 (±1.1)	2.7 (±0.7)	0.87
	21	6.2 (±1.3)	6.3 (±1.3)	0.84

*: Only 12 pairs analyzed for total IgE.

## Discussion

This is the first report of the effect of concomitant intestinal parasitic infection on the human immune response to severe infection with *V. cholerae*. The major observation of this study is that helminth-infected cholera patients have markedly decreased mucosal immune responses to the immunodominant *V. cholerae* protein antigen, CTB. In contrast, helminth- infected patients responded as well as non-infected patients to the polysaccharide antigen LPS, measured by both direct ELISA and by their vibriocidal antibody response – the complement activating antibody associated with protective immunity to cholera. This difference in immune responses to CTB was highly statistically significant and was observed independently for patients with three major types of helminth infection including *Ascaris*, *Trichuris*, and hookworm infections. The difference was not observed in intestinal protozoal infection. Furthermore, this observation was independent of potential confounders including age and markers of nutritional status.

What explains the difference in the response of helminth-infected patients to CTB but not LPS? One possibility is that this restricted finding is related to immunomodulatory effects of helminth infection that are specific to CD4+ T-cell responses to *V. cholerae*. In a mouse model of the *V. cholerae* immune response, humoral responses to the protein antigen CTB are dependent on an intact population of CD4+ T-cells, while the LPS humoral immune response evolves in a T-cell independent mechanism, and is equally robust in CD4+ T-cell depleted and non-CD4+ depleted mice [23],[24]. Similarly, in human *V. cholerae*, humoral LPS responses may also occur primarily in a T-cell independent manner. Thus, the differential effect of helminth infection on the CTB and LPS immune responses may implicate CD4+ T cells as a target of helminthic immunomodulation.

Previous immunologic studies, primarily using mouse models of concomitant enteric helminth and bacterial infection, demonstrate the importance of T-cell dependent immunomodulation by helminth infection. Furthermore, these studies suggest that helminth infection may result in a relative increased shift in the Th1/Th2 polarization of the immune response, with concomitant helminth infection favoring a predominantly Th2-type driven immune response and a decrease in Th1-type immune response [25],[26]. Although we similarly hypothesized that helminth infected cholera patients may have Th2>Th1 polarized immune responses, with class switch recombination favoring intact responses of IgE and IgG4 antibodies and relatively diminished IgG1 and IgG3 responses [27], only IgG1 was significantly different between helminth infected and non-infected individuals. Thus, although the differential effect of helminth infection on the CTB and LPS immune response suggests a possible CD4+ T-cell dependent mechanism, our IgG subclass studies suggest that the immunomodulatory effect of concomitant parasitic infection on the immune response to the mucosal pathogen *V. cholerae* does not fall easily into a classic Th1 or Th2 polarization of the immune response.

Instead, our data suggest that the immunomodulating effect of helminth infection on the humoral immune response to the mucosal pathogen *V. cholerae* results predominantly in an overall decrease in the mucosal antibody response. This hypothesis is supported by our observation that non-helminth infected patients demonstrated a much greater ability to mount a rapid (possibly anamnestic) fecal CTB-specific IgA response on day 2 after the onset of illness, and that ultimately these non-helminth infected patients had almost a 2 fold greater increase in their convalescent serum CTB IgA antibody.

Our study has some notable limitations. The sensitivity of direct microscopic examination for stool parasites in the setting of acute watery diarrhea is not well characterized. Theoretically, the presence of acute watery diarrhea may decrease the sensitivity of the stool examination for parasites. In a study by Goodman and colleagues, no significant association between diarrheal stool and the sensitivity of detection of helminth infections using a direct sedimentation technique was observed overall, though diarrheal stool did result in diminished sensitivity for *Trichuris* eggs [28]. Although acute cholera may result in some false negative tests for stool helminths, this type of misclassification is most likely to introduce a bias that would decrease our ability to detect a difference between helminth infected and non-infected cholera patients. Despite this possibility, we still found robust, consistent differences in the CTB immune response across these groups.

Another limitation of this observational study is that causal relationships are indeterminate. Although we present hypotheses to account for the difference in the immune response between helminth infected and non-infected cholera patients, our observational data falls outside the expected model where concomitant helminth infection results primarily in a Th2 shifted response to bacterial co-infection. However, the finding that helminth infection has a profound association with decreased mucosal humoral immune responses to *V. cholerae* infection carries implications for the development of protective immunity in cholera endemic areas and may provide an additional rational basis for deworming programs in cholera endemic areas. Additional studies, including further characterization of the role of T cells in the immune response to human *V. cholerae* infection, and the development of an animal model of co-infection with intestinal helminths and *V. cholerae* might provide additional insight into the mechanisms underlying these findings.

## References

[1] Zuckerman JN, Rombo L, Fisch A (2007). The true burden and risk of cholera: Implications for prevention and control.. Lancet Infect Dis.

[2] Ryan ET, Dhar U, Khan WA, Salam MA, Faruque AS (2000). Mortality, morbidity, and microbiology of endemic cholera among hospitalized patients in dhaka, bangladesh.. Am J Trop Med Hyg.

[3] Chowdhury F, Khan AI, Harris JB, Larocque RC, Chowdhury MI (2008). A comparison of clinical and immunologic features in children and older patients hospitalized with severe cholera in Bangladesh.. Pediatr Infect Dis J.

[4] Northrop-Clewes CA, Rousham EK, Mascie-Taylor CN, Lunn PG (2001). Anthelmintic treatment of rural bangladeshi children: Effect on host physiology, growth, and biochemical status.. Am J Clin Nutr.

[5] Saha DR, Rajendran K, Ramamurthy T, Nandy RK, Bhattacharya SK (2008). Intestinal parasitism and Vibrio cholerae infection among diarrhoeal patients in Kolkata, India.. Epidemiol Infect.

[6] Tamang MD, Sharma N, Makaju RK, Sarma AN, Koju R (2005). An outbreak of El Tor cholera in Kavre district, Nepal.. Kathmandu Univ Med J (KUMJ).

[7] Ljungstrom I, Holmgren J, Huldt G, Lange S, Svennerholm AM (1980). Changes in intestinal fluid transport and immune responses to enterotoxins due to concomitant parasitic infection.. Infect Immun.

[8] Holmgren J, Svennerholm AM (1977). Mechanisms of disease and immunity in cholera: a review.. J Infect Dis.

[9] Mosley WH, Ahmad S, Benenson AS, Ahmed A (1968). The relationship of vibriocidal antibody titre to susceptibility to cholera in family contacts of cholera patients.. Bull World Health Organ.

[10] Glass RI, Svennerholm AM, Khan MR, Huda S, Huq MI (1985). Seroepidemiological studies of El Tor cholera in Bangladesh: association of serum antibody levels with protection.. J Infect Dis.

[11] Saha D, LaRocque RC, Khan AI, Harris JB, Begum YA (2004). Incomplete correlation of serum vibriocidal antibody titer with protection from Vibrio cholerae infection in urban Bangladesh.. J Infect Dis.

[12] Lycke N (1997). The mechanism of cholera toxin adjuvanticity.. Res Immunol.

[13] Harris JB, Larocque RC, Chowdhury F, Khan AI, Logvinenko T (2008). Susceptibility to vibrio cholerae infection in a cohort of household contacts of patients with cholera in Bangladesh.. PLoS Negl Trop Dis.

[14] Levine MM, Kaper JB, Herrington D, Ketley J, Losonsky G (1988). Safety, immunogenicity, and efficacy of recombinant live oral cholera vaccines, CVD 103 and CVD 103-HgR.. Lancet.

[15] Richie EE, Punjabi NH, Sidharta YY, Peetosutan KK, Sukandar MM (2000). Efficacy trial of single-dose live oral cholera vaccine CVD 103-HgR in North Jakarta, Indonesia, a cholera-endemic area.. Vaccine.

[16] Tacket CO, Cohen MB, Wasserman SS, Losonsky G, Livio S (1999). Randomized, double-blind, placebo-controlled, multicentered trial of the efficacy of a single dose of live oral cholera vaccine CVD 103-HgR in preventing cholera following challenge with vibrio cholerae O1 el tor inaba three months after vaccination.. Infect Immun.

[17] Cooper PJ, Chico ME, Losonsky G, Sandoval C, Espinel I (2000). Albendazole treatment of children with ascariasis enhances the vibriocidal antibody response to the live attenuated oral cholera vaccine CVD 103-HgR.. J Infect Dis.

[18] Cooper PJ, Chico M, Sandoval C, Espinel I, Guevara A (2001). Human infection with ascaris lumbricoides is associated with suppression of the interleukin-2 response to recombinant cholera toxin B subunit following vaccination with the live oral cholera vaccine CVD 103-HgR.. Infect Immun.

[19] Rahman M, Sack DA, Mahmood S, Hossain A (1987). Rapid diagnosis of cholera by coagglutination test using 4-h fecal enrichment cultures.. J Clin Microbiol.

[20] Qadri F, Azim T, Chowdhury A, Hossain J, Sack RB (1994). Production, characterization, and application of monoclonal antibodies to Vibrio cholerae O139 synonym bengal.. Clin Diagn Lab Immunol.

[21] Qadri F, Mohi G, Hossain J, Azim T, Khan AM (1995). Comparison of the vibriocidal antibody response in cholera due to Vibrio cholerae O139 Bengal with the response in cholera due to Vibrio cholerae O1.. Clin Diagn Lab Immunol.

[22] Qadri F, Ryan ET, Faruque AS, Ahmed F, Khan AI (2003). Antigen-specific immunoglobulin A antibodies secreted from circulating B cells are an effective marker for recent local immune responses in patients with cholera: comparison to antibody-secreting cell responses and other immunological markers.. Infect Immun.

[23] Kately JR, Patel CB, Friedman H (1975). Involvement of T- and B-lymphocytes in the immune response to the protein exotoxin and the lipopolysaccharide antigens of Vibrio cholerae.. Ann N Y Acad Sci.

[24] Hornqvist E, Goldschmidt TJ, Holmdahl R, Lycke N (1991). Host defense against cholera toxin is strongly CD4+ T cell dependent.. Infect Immun.

[25] Nagler-Anderson C (2000). Tolerance and immunity in the intestinal immune system.. Crit Rev Immunol.

[26] Weng M, Huntley D, Huang IF, Foye-Jackson O, Wang L (2007). Alternatively activated macrophages in intestinal helminth infection: effects on concurrent bacterial colitis.. J Immunol.

[27] Garraud O, Perraut R, Riveau G, Nutman TB (2003). Class and subclass selection in parasite-specific antibody responses.. Trends Parasitol.

[28] Goodman D, Haji HJ, Bickle QD, Stoltzfus RJ, Tielsch JM (2007). A comparison of methods for detecting the eggs of Ascaris, Trichuris, and hookworm in infant stool, and the epidemiology of infection in Zanzibari infants.. Am J Trop Med Hyg.

